# Obsessive–compulsive symptoms in young women affected with anorexia nervosa, and their relationship with personality, psychopathology, and attachment style

**DOI:** 10.1007/s40519-021-01252-y

**Published:** 2021-06-30

**Authors:** Federico Amianto, Ilaria Secci, Luca Arletti, Chiara Davico, Giovanni Abbate Daga, Benedetto Vitiello

**Affiliations:** 1 Neurosciences Department, University of Turin, Via Cherasco 11, 10126 Turin, Italy; 2 Department of Public Health and Pediatric Sciences, Section of Child and Adolescent Neuropsychiatry, University of Turin, P.zza Polonia 94, 10126 Turin, Italy

**Keywords:** Anorexia nervosa, Obsessive–compulsive symptoms, Y-BOCS, Attachment style, Personality traits, Phobic anxiety

## Abstract

**Purpose:**

Obsessive–compulsive symptoms (OC) are associated with greater morbidity and worse prognosis in anorexia nervosa (AN). We assessed the presence of non-eating OC in participants with AN and related them with their psychopathology, personality, and attachment style features.

**Methods:**

Young women with AN (*N* = 41, 30 restrictor and 11 binge-purging type) were assessed on the Yale-Brown Obsessive–Compulsive Scale (Y-BOCS). These participants with AN and 82 healthy controls (HC) completed the Temperament and Character Inventory (TCI), Eating Disorder Inventory-2 (EDI-2), Symptom Checklist-90 (SCL-90), Toronto Alexithymia Scale (TAS-20), and Attachment Style Questionnaire (ASQ). The association between Y-BOCS scores and indexes of psychopathology, personality, and attachment were examined.

**Results:**

AN had significantly higher scores than HC on the EDI-2, SCL-90, TAS-20, ASQ-Need for Approval, and TCI-Harm Avoidance and Self-directedness. The Y-BOCS scores were significantly correlated with ASQ-Need for Approval, TAS-20-Difficulty in Describing Feelings, SCL-90-Phobic Anxiety, and Anxiety, EDI-2-Drive to Thinness, and Asceticism. Need for Approval displayed the strongest correlation with OC symptoms. Difficulty in describing feelings displayed the strongest correlation with compulsive OC symptoms.

**Conclusions:**

OC traits in AN were primarily associated with measures of insecure attachment rather than to their eating disorder or general psychopathology. Therapeutic approaches to correcting insecure attachment may be considered as a possible approach to treating AN patients with OC. The study supports a new psychopathological perspective for understanding the meaning of OC symptoms in AN.

**Level of evidence:**

III: Evidence obtained from cohort or case–control analytic studies.

## Introduction

An association between obsessive–compulsive features (OC) and eating disorders (EDs), in particular anorexia nervosa (AN), has long been reported [1–5]. Some authors raised the possibility of a psychogenetic connection between the two disorders through the involvement of the serotonergic system [6–8]. Moreover, the inclusion of AN into a broad obsessive–compulsive disorder (OCD) spectrum has been proposed [9, 10].

There are indeed analogies in the symptomatic manifestations of AN and OCD, with the presence of intrusive and distressing thoughts accompanied by the urge to implement compulsive behaviors to reduce the anxiety generated by the obsessions [11–13]. In AN, behaviors such as food restriction, exercise and vomiting are often accompanied by rigid rituals, such as eating food according to a certain order, calculating the caloric content of each food ingested, or chewing food a precise number of times [14]. Often in OCD there are rituals related to food, sometimes leading to weight loss [15, 16]. In both disorders the OC tendencies may serve a similar function of affective regulation [17]. For these reasons clinicians may find it difficult to recognize the nature of the observed OC in AN and to distinguish between behaviors that are primarily eating disorder symptoms and those that are expression of a comorbid OCD. Moreover, it is unclear if food-related OC symptoms share a similar pathogenesis with the non-food-related OC symptoms, or if they should be distinguished and treated with different approaches. A better comprehension of the relationship between the most common psychopathologic features of AN and non-food-related OC symptoms may help clinicians in the management of this comorbidity.

The prevalence of OCD in subjects with EDs has been reported to vary from 10 to 60%, significantly higher than the 1–2% rate found in the general population [18, 19]. In subjects suffering from OCD, on the other hand, there is a 10–17% risk of being affected by EDs [20, 21].

A link between these disorders may be their relationship with the anxiety disorders’ spectrum. In DSM-IV, OCD was classified among the anxiety disorders [22]. The AN is also characterized by high levels of anxiety with respect to food intake and weight increase [23, 24]. The quality of anxiety in both disorders may be atypical with respect to other anxiety disorders, since the use of the benzodiazepines has a limited effect in both OCD and EDs [25]. Moreover, although anxiety disorders and OCD are highly prevalent among women with AN, only OCD is a predictor of developing AN [26].

Another point of junction is represented by personality characteristics. Both OCD and AN display a higher prevalence of cluster C personality disorders [27, 28]. The rate of obsessive–compulsive personality disorder (OCPD) was found to be 23–35% in OCD, compared to 1–2% in the general population [29, 30]. In restrictor-type AN, OCPD represents the most frequent personality disorder with a prevalence similar to that in OCD [28]. OC traits, such as perfectionism, inflexibility, and the need for order, were also found in the offspring of participants with AN with OCD, regardless of a comorbid OCPD [31]. In terms of personality traits, both OCD and AN are characterized by low novelty seeking, high harm avoidance, high persistence and low self-directedness [32]. The presence of OC traits in childhood, such as perfectionism and the need for order, was associated with a greater rate of comorbid OCD and AN in adulthood [33].

The comorbidity between AN and OCD represents a therapeutic challenge: it is associated with a greater severity of eating symptoms, a tendency to physical hyperactivity, a higher rate of anxiety and depression, and a higher probability of relapse [4, 34–36]. On the other hand, the malnutrition that characterizes AN has important cognitive effects, which can worsen the obsessive symptoms and influence the response to therapies for OCD [37].

Despite the above-mentioned, well-documented links between AN and OCD, the relationship of OC symptoms with the psychopathological roots of AN remains unexplored. Are OC symptoms related to particularly severe anxiety comorbidity? Are they a collateral symptomatic expression of highly malfunctioning personality traits? Do they strictly relate to the eating psycopathology? Since impaired attachment and alexithymic traits are relevant to the pathogenesis of AN [38], OC symptoms might be related to these psychopathological features which had not been taken into account in previous studies.

This paper seeks to answer the aforementioned unsolved questions concerning the presence of OC symptoms in a clinical group of patients diagnosed with AN. In particular, it was investigated whether the non-eating obsessive symptoms detected in participants with AN correlated with eating psychopathology, personality traits, or attachment style as rated with psychological tests. The profile of participants with AN was compared to that of healthy controls (HC), and possible association of symptoms of OC with personality and psychopathology characteristics in AN were examined.

## Methods

### *Sample*

Participants were recruited from the University of Turin Regional Pilot Center for Eating Disorders, in Turin (Italy), which provides inpatient, day hospital, and outpatient services. All the patients who were in treatment at the center between June 2018 and June 2019 were considered. Participants received a psychiatric examination to determine the presence of AN using the Structured Clinical Interview for Diagnosis (SCID) for DSM-IV-TR, a tool that has fair to excellent inter-rater reliability on axis I and excellent on axis II diagnoses [39].

In order to make the sample as homogeneous as possible and increase study specificity, we adopted strict inclusion and exclusion criteria. In particular, entry criteria included the following: (1) a full diagnosis of AN according to the SCID; (2) female sex only, to avoid sex-related differences; (3) BMI ≥ 14, to avoid severe malnutrition, which has been shown to affect brain functioning; and (4) Caucasian of Italian origin or UE origin with mother-language knowledge of Italian language. Excluded were subjects with: (1) intellectual disability; (2) developmental or learning disorders; (3) acute psychotic disorders; (4) neurological disorder (e.g., multiple sclerosis, stroke, history of severe head trauma); or (5) substance abuse.

From an initial group of 54 recruited subjects with AN, 13 were excluded (8 for BMI below 14, 2 for failure to complete the tests, and 3 for male gender). The final group consisted of 41 participants with AN (30 restrictive and 11 binge-purging type), aged between 16 and 30 years (Table 1). Among these, 8 (19%) received a clinical diagnosis of OCD during the clinical assessment.

**Table 1:** Clinical and demographic data

	Participants with AN (*n* = 41)	Controls (*n* = 82)	t	*P*	*Cohen’s D*
	mn ± sd	mn ± sd			
BMI	16.55 ± 2.07	21.20 ± 2.32	10.788	0.001	− 2.081
CGI score	5.07 ± 0.60	–	–	–	–
Age of onset (years)	15.93 ± 3.17	–	-	–	–
Age (years)	21.03 ± 6.12	23.13 ± 0.85	2.535	0.013	− 0.525
Binge-eating/week	1.46 ± 4.17	–	–	–	–

*AN* anorexia nervosa, *mn * mean, *sd * standard deviation, *BMI* body mass index, *CGI * clinical global impression

### Description of controls

A group of HC randomly selected from the database of the Neurosciences Department of the University of Turin was included for the present study. The comparison to the controls was conducted to document the overall consistence of the participants with AN with respect to previous literature [23, 32, 40, 41] and to select the variables to be correlated with Y-BOCS scores. The database was composed of healthy subjects (university student volunteers recruited after curricular lessons) who were screened for major psychiatric disorders using the SCID, and then assessed with the same self-administered instruments applied to the AN participants. The included control sample consisted of 82 healthy female controls (HC), aged between 22 and 24 years and matched with the AN participants on sociocultural status. Students were informed about the purpose of the study and obtained assurance about their anonymity. Written informed consent was obtained from each student before test delivery.

The HC did not receive the Y-BOCS administration because they belonged to a previously recruited cohort. We chose not to recruit a HC group to be assessed with Y-BOCS and self-rated scales in consideration of the results in the previous literature which indicated very low Y-BOCS scores in the normal population [43] and thus suggested the impossibility to perform a consistent correlation analysis between the Y-BOCS scores and psychopathological measures in the healthy subjects.

### Ethics

All participants provided written informed consent prior to be included in the study and assessed. This study was performed in accordance with the 1995 Declaration of Helsinki, as revised in Edinburgh, in October 2000. The students’ recruitment and assessment were approved by the Bioethics Committee of the University of Turin, Italy (Protocol Number: 127252).

In agreement with the Inter-Hospital Ethical Committee (CEI), the authorization was not requested for clinical participants since all procedures were part of the routine clinical procedures.

### Hetero-administered interview for OC symptoms

All participants with AN were given the *Yale-Brown Obsessive–Compulsive Scale* (Y-BOCS) [42], a scale designed for use as a semi-structured interview for assessing type and severity of OC symptoms. The interviewer assesses the presence and severity of OC symptoms in the last week (including the time of the interview). Before proceeding with the interview, participants with AN were provided with a clear definition of the concepts of obsessions and compulsions. The Y-BOCS administration requires interviewer training before administration to the patient. One of the authors (IS) underwent training in this instrument. The Y-BOCS scale was administered with a blind procedure with respect to the results of the self-administered inventories and clinical assessment, i.e., the interviewer was not informed about the results of the self-administered inventories at the moment of the administration of the interview.

The interview produces results based on 0–4 Likert scales. The results are articulated in six scales. Three are clinical scales: Obsessions (O), Compulsions (C), Total Score (O + C), and three are qualitative scales: Global Severity, Insight and Reliability. The total Y-BOCS score displays 5 levels of severity: 0–7 subclinical; 8–15 mild; 16–23 moderate; 24–31 severe; and 32–40 extreme.

### Self-administered inventories

AN and HC completed a battery of self-administered psychometric tests assessing personality and psychopathology features, as well as attachment style. The battery included the following:

The *temperament and character inventory* (TCI) [44]: a questionnaire composed of 240 items that investigate seven personality dimensions distinguished in 4 dimensions of the temperament and 3 of the character, according to the neurobiological model proposed by Cloninger.

The temperamental traitsinclude the following: Harm avoidance (HA), Novelty Seeking (NS), Reward Dependence (RD), and Persistence (P). High scores in the temperament traits represent extremely high expression of the trait, and low scores low expression of the trait. The character dimensions include the following: Self-directedness (SD), Cooperativeness (C), Self-transcendence (ST). High scores in the character traits represent character development, low scores low character development. (Chronbach’s alpha of the Italian version = 0.72).

The *Eating Disorder Inventory-2* (EDI-2) [45] is a questionnaire that evaluates the psychopathological characteristics salient of eating disorders. These features were formulated in 91 items and divided into the following 11 subscales: Drive to thinness (DT), Bulimia (BU), Body Dissatisfaction (BD), Inadequacy (IN), Perfectionism (P), Interpersonal distrust (ID), Enteroceptive awareness (EA), Fear of maturity (MF), Ascetism (ASC), Impulsiveness (I), Social insecurity (SI). High scores represent greater levels of psychopathology. (Chronbach’s alpha of the Italian version = 0.81).

The *Toronto Alexithymia Scale* (TAS-20) [46] is a questionnaire of 20 items, used to assess the level of alexithymia. It is divided into three following factorial scales: 1. difficulty in identifying feelings; 2. difficulty in describing feelings; and 3. thought oriented towards the outside: cognitive style polarized towards the meticulous analysis of external reality. The total TAS-20 score, obtained by adding the scores for the three subscales, is between 20 and 100, with a cut-off of 61. High scores represent greater levels of psychopathology. (Chronbach’s alpha of the Italian version = 0.72).

The *Symptom Checklist-90 SCL-90* (SCL-90) [47] is a Test composed of 90 items that assesses the presence and severity of symptoms of mental illness in different symptom domains during the last week (including the day of the evaluation). The subject's responses are interpreted on the basis of nine primary symptom dimensions, which are as follows: Somatization (SOM), Obsessivity–Compulsivity (O-C), Interpersonal hypersensitivity (IS), Depression (DEP), Anxiety (ANX), Hostility (HOS), Phobic anxiety (PHOB), Paranoid ideation (PAR), Psychoticism (PSY). A tenth dimension, the Total Score (TOT), is the sum of the previous ones. High scores represent greater levels of psychopathology. (Chronbach’s alpha of the Italian version = 0.96).

The *Attachment Style Questionnaire* (ASQ) [48] is a self-administered questionnaire, composed of 40 items evaluated through a 6-point scale (1 totally disagree, 6 totally agreed), used to identify the style of attachment within interpersonal relationships. Analyzing the main components of the questionnaire based on the Bartholomew model, the 40 items were divided into 5 scales as follows: Trust (8 item) that defines the safe style, Discomfort due to intimacy (10 items), Secondariness of relations (8 items) (These last two items define an avoidant/detached style), Need for approval (7 items), Concern for relationships (7 items) (These last two items define a worried or anxious style). Low scores in Trust and high scores in the other four dimensions represent insecure attachment. (Chronbach’s alpha of the Italian version = 0.79).

### Data analysis

The Y-BOCS total score obtained from the participants with AN was analyzed comparing the subgroup with a clinical diagnosis of OCD with the subgroup with OCD. The Mann–Whitney *U* test, a nonparametric test, was used given the absence of normality with a small sample size of the subgroup with OCD (*N* = 8).

The total Y-BOCS score of AN participants was compared to that of a healthy population extracted from the literature, using the Student’s *t* test [43]. The distribution of the patient’s sample in the five Y-BOCS categories was examined. A within-sample ANOVA comparison of Y-BOCS sub-scores and total scores was made between the three subgroups of outpatients with AN, inpatients with AN in ordinary admission, and DH inpatients with AN, to identify possible differences related to the clinical status. A comparison between participants with AN and HC was performed using Student's t-tests for clinical, demographic, and psychometric measures. The ANCOVA was applied to compare the psychometric variables among groups using the demographic variables which resulted significantly different between groups (i.e., BMI and age). In consideration of the high number of comparisons, a correction for the *p* value was adopted to reduce type I errors, with a *p* < 0.001 value being accepted for significance.

A Pearson’s correlation analysis was performed between the Y-BOCS scores of the participants with AN, and their BMI, age of onset of the disease, and those psychometric variables which differed between participants with AN and HC. In consideration of the use of the *p* correction in the previous analyses to account for multiple comparisons, and because of the explorative aims of the study, a more conservative correction was applied for the correlation analysis, with a *p* < 0.01 value accepted as significant.

The statistical software package Statistical Package for Social Sciences SPSS 27.0 was used for the data analyses.

## Results

### Comparison of Y-BOCS score among AN clinical subgroups and between participants with AN and healthy population

Participants with AN with a clinical diagnosis of OCD displayed a higher Y-BOCS total score (31.50 ± 4.75 vs 18.70 ± 7.27; *U* = 251,00; *p* < 0.001); higher obsessions (15.63 ± 2.87 vs 9.82 ± 4.36; *U* = 229,00; *p* < 0.001); higher compulsions (15.88 ± 3.31 vs 8.88 ± 4.20; *U* = 242,00; *p* < 0.001); and higher severity scores (4.75 ± 0.46 vs 2.03 ± 1.32; *U* = 260,00; *p* < 0.001).

Participants with AN displayed a mean total Y-BOCS score which was in the moderate range (mean = 21.20, SD = 8.5) and thus was higher than that of the healthy population as obtained from the literature (mean = 0.31, SD = 1.21; *t* = 5.34; *p* < 0.001) [43].

### Sociodemographic and clinical features in participant groups

Table 1 displays the comparison between participants’ groups. The average BMI of participants with AN was lower than that of HC (*p* < 0.001). The average age of the participants with AN was lower than HC (*p* < 0.010).

### Y-BOCS sub-scores distribution among participants with AN and clinical subgroups comparison

**Fig. 1:**
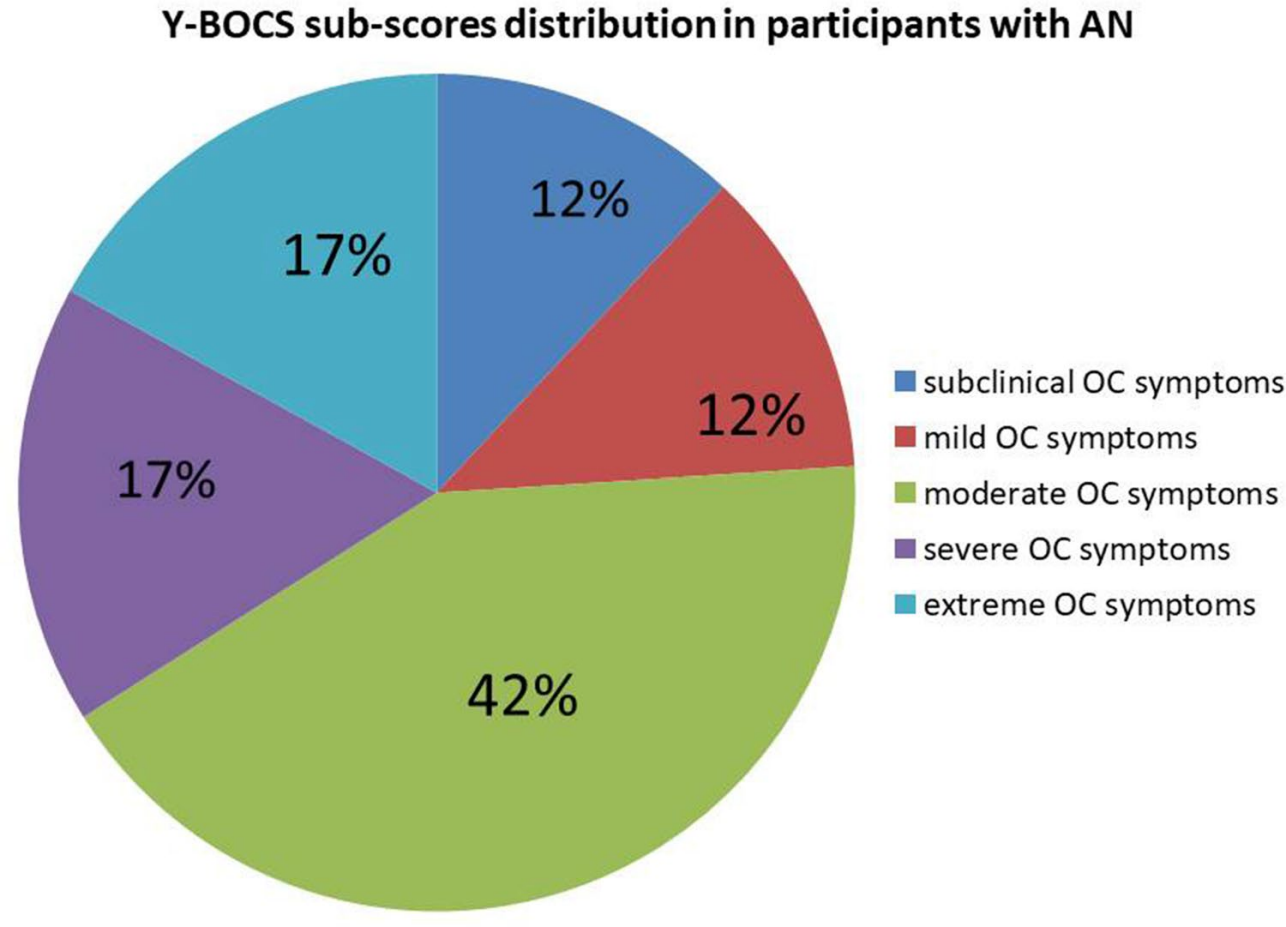
Y-BOCS sub-scores distribution among participants with AN. The figure presents the distribution of the degrees of severity of OC symptoms in the group of participants with AN. The following five degrees of severity are recognized by the Y-BOCS scale: subclinical, mild, moderate, severe, and extreme. The number indicates the percentage of subjects in each subgroup

The distribution of participants with AN among the classes of symptom severity (Fig. 1) was the following: 12% of participants with AN fell into the subclinical severity category, 12% manifested mild symptoms, 42% symptoms of moderate severity, 17% severe symptoms and 17% symptoms of extreme degree. No statistically significant differences were observed in the Y-BOCS scores among the treatment subgroups.

### ANCOVA comparison between participants with AN and HC

As shown in Table 2, participants with AN display higher HA (*p* < 0.001) and lower SD (*p* < 0.001). Participants with AN displayed higher scores in all EDI-2 subscales (*p* < 0.001), in all SCL-90 scores (*p* < 0.001), in difficulty in identifying feelings, difficulty in describing feelings, and in TAS-20 total score (*p* < 0.001), compared to HC. Participants with AN scored significantly higher than the controls also in the need for approval subscale of the ASQ (*p* < 0.001).

**Table 2:** Comparison of personality and psychopathology variables: significant differences

	Participants with AN (*n* = 41)	Controls (*n* = 82)	*F*	*P*
	mn ± sd	mn ± sd		
Novelty Seeking (TCI)	16.62 ± 8.88	18.31 ± 5.44	0.774	0.464
Harm Avoidance (TCI)	29.32 ± 18.25	18.53 ± 7.37	8.560	**0.001**
Reward Dependence (TCI)	14.32 ± 5.17	15.33 ± 4.03	1.280	0.283
Persistence (TCI)	7.59 ± 12.62	4.66 ± 2.22	1.429	0.245
Self-Directedness (TCI)	20.38 ± 8.96	28.30 ± 8.75	12.479	**0.001**
Cooperativeness (TCI)	28.92 ± 9.00	30.60 ± 7.62	1.580	0.212
Self-Trascendence (TCI)	11.57 ± 7.33	12.03 ± 7.24	0.571	0.567
Drive to thinness (EDI-2)	14.97 ± 6.16	2.02 ± 4.18	86.540	**0.001**
Bulimia (EDI-2)	3.45 ± 5.18	1.48 ± 3.00	3.451	0.036
Body Dissatisfaction (EDI-2)	14.66 ± 7.71	5.56 ± 7.08	15.566	**0.001**
Inadequacy (EDI-2)	16.18 ± 9.82	3.31 ± 4.86	35.733	**0.001**
Perfectionism (EDI-2)	7.24 ± 3.72	4.86 ± 3.68	4.149	0.019
Interpersonal Distrust (EDI-2)	10.61 ± 5.61	3.36 ± 3.60	29.663	**0.001**
Interoceptive Awareness (EDI-2)	17.03 ± 9.30	2.19 ± 3.09	64.459	**0.001**
Maturity Fears (EDI-2)	9.97 ± 6.35	3.97 ± 4.98	11.224	**0.001**
Asceticism (EDI-2)	10.03 ± 4.95	3.37 ± 2.75	32.154	**0.001**
Impulsivity (EDI-2)	10.61 ± 8.47	1.99 ± 3.22	22.892	**0.001**
Social Insecurity (EDI-2)	9.97 ± 4.26	3.95 ± 3.71	24.465	**0.001**
Somatization (SCL-90)	22.84 ± 11.11	7.03 ± 6.03	57.990	**0.001**
Obsessive-compulsivity (SCL-90)	23.88 ± 10.13	8.56 ± 5.62	57.224	**0.001**
Interpersonal Oversensibility (SCL-90)	20.19 ± 9.44	7.02 ± 5.84	41.671	**0.001**
Depression (SCL-90)	31.12 ± 12.73	9.97 ± 7.75	59.891	**0.001**
Anxiety (SCL-90)	22.46 ± 9.87	7.21 ± 4.92	60.199	**0.001**
Hostility (SCL-90)	9.77 ± 7.33	3.06 ± 2.49	28.388	**0.001**
Phobic Anxiety (SCL-90)	9.38 ± 6.78	1.84 ± 2.01	41.339	**0.001**
Paranoid Ideation (SCL-90)	10.08 ± 5.96	4.33 ± 4.13	16.171	**0.001**
Psycoticism (SCL-90)	13.38 ± 8.04	3.98 ± 3.49	39.251	**0.001**
SCL-90 total score	170.27 ± 82.06	57.03 ± 34.86	55.558	**0.001**
Confidence (ASQ)	30.11 ± 29.66	30.87 ± 4.85	0.260	0.772
Discomfort with Closeness (ASQ)	28.40 ± 7.14	27.05 ± 6.29	0.526	0.593
Relationships as Secondary (ASQ)	36.81 ± 5.30	33.86 ± 5.95	4.764	0.011
Need for approval (ASQ)	29.59 ± 6.06	22.65 ± 5.35	16.944	**0.001**
Concern for Relationships (ASQ)	21.30 ± 5.82	19.25 ± 5.69	0.772	0.465
Difficulty in Identifying Feelings (TAS-20)	24.78 ± 5.26	11.99 ± 5.43	59.321	**0.001**
Difficulty in Describing Feelings (TAS-20)	18.50 ± 5.10	12.87 ± 5.52	12.836	**0.001**
Externally-Oriented Thinking (TAS-20)	19.14 ± 4.56	17.22 ± 5.42	1.305	0.276
Alexithimia (total score) (TAS-20)	61.36 ± 12.00	42.17 ± 9.33	36.933	**0.001**

Accepted significance *p* < .001. Mn = mean; sd = standard deviation. Bold characters indicate the variables which are included in the next analysis

Accepted significance p < .001 is evidenced in bold characters which indicate the variables which are included in the next analysis

### Pearson's linear correlation between Y-BOCS, and personality and psychopathology features

The variables which differed between participants with AN and HC were correlated with Y-BOCS scores among the participants with AN. Table 3 displays only the significant correlations observed between Y-BOCS scores and the other features. The total Y-BOCS score positively correlated with need for approval (*p* < 0.007) and difficulty in describing feelings (*p* < 0.007). The Y-BOCS subtotal score “obsessions” positively correlated with need for approval (*p* < 0.002), anxiety (*p* < 0.003) and phobic anxiety (*p* < 0.002). The Y-BOCS subtotal score “compulsions” positively correlated with difficulty in describing feelings (*p* < 0.002).

**Table 3:** Pearson correlation between Y-BOCS scores, personality and psychopathology

Y-BOCS variable	Correlated variable	*r*	*P*
Y-BOCS total score	Harm Avoidance (TCI)	− 0.029	0.863
	Self-Directedness (TCI)	− 0.084	0.622
	Drive to Thinness (EDI-2)	0.392	0.015
	Body Dissatisfaction (EDI-2)	0.198	0.234
	Inadequacy (EDI-2)	0.139	0.404
	Interpersonal Distrust (EDI-2)	0.181	0.277
	Interoceptive Awareness (EDI-2)	0.210	0.206
	Maturity Fears (EDI-2)	− 0.137	0.413
	Asceticism (EDI-2)	0.367	0.023
	Impulsivity (EDI-2)	0.310	0.058
	Social Insecurity (EDI-2)	0.221	0.183
	Somatization (SCL-90)	0.255	0.219
	Obsessive-Compulsivity (SCL-90)	0.214	0.294
	Interpersonal Over sensibility (SCL-90)	0.215	0.290
	Depression (SCL-90)	0.349	0.080
	Anxiety (SCL-90)	0.419	0.033
	Hostility (SCL-90)	0.184	0.369
	Phobic Anxiety (SCL-90)	0.492	0.011
	Paranoid Ideation (SCL-90)	0.052	0.802
	Psychoticism (SCL-90)	0.183	0.372
	SCL-90 total score	0.237	0.243
	Need for Approval (ASQ)	0.433	**0.007**
	Difficulty in Identifying Feelings (TAS-20)	0.196	0.253
	Difficulty in describing feelings (TAS-20)	0.444	**0.007**
	Alexithymia (total score) (TAS-20)	0.250	0.142
Y-BOCS subtotal obsessions	Harm Avoidance (TCI)	0.060	0.722
	Self-Directedness (TCI)	− 0.278	0.095
	Drive to Thinness (EDI-2)	0.342	0.036
	Body Dissatisfaction (EDI-2)	0.259	0.116
	Inadequacy (EDI-2)	0.221	0.183
	Interpersonal Distrust (EDI-2)	0.184	0.268
	Interoceptive Awareness (EDI-2)	0.225	0.175
	Maturity Fears (EDI-2)	0.065	0.699
	Asceticism (EDI-2)	0.352	0.030
	Impulsivity (EDI-2)	0.309	0.059
	Social Insecurity (EDI-2)	0.277	0.092
	Somatization (SCL-90)	0.415	0.039
	Obsessive-Compulsivity (SCL-90)	0.428	0.029
	Interpersonal Over-sensibility (SCL-90)	0.328	0.102
	Depression (SCL-90)	0.479	0.013
	Anxiety (SCL-90)	0.560	**0.003**
	Hostility (SCL-90)	0.197	0.334
	Phobic anxiety (SCL-90)	0.590	**0.002**
	Paranoid Anxiety (SCL-90)	0.152	0.459
	Psychoticism (SCL-90)	0.324	0.107
	SCL-90 total score	0.329	0.101
	Need for Approval (ASQ)	0.495	**0.002**
	Difficulty in Identifying Feelings (TAS-20)	0.089	0.606
	Difficulty in describing feelings (TAS-20)	0.284	0.093
	Alexthymia (total score) (TAS-20)	0.091	0.596
Y-BOCS subtotal compulsions	Harm Avoidance (TCI)	− 0.106	0.534
	Self-Directedness (TCI)	0.118	0.487
	Drive to Thinness (EDI-2)	0.341	0.036
	Body Dissatisfaction (EDI-2)	0.091	0.588
	Inadequacy (EDI-2)	0.028	0.866
	Interpersonal Distrust (EDI-2)	0.132	0.429
	Interoceptive Awareness (EDI-2)	0.144	0.389
	Maturity Fears (EDI-2)	− 0.290	0.077
	Asceticism (EDI-2)	0.290	0.078
	Impulsivity (EDI-2)	0.234	0.158
	Social Insecurity (EDI-2)	0.113	0.499
	Somatization (SCL-90)	0.047	0.824
	Obsessive-Compulsivity (SCL-90)	− 0.040	0.845
	Interpersonal Over-sensibility (SCL-90)	0.059	0.775
	Depression (SCL-90)	0.146	0.478
	Anxiety (SCL-90)	0.190	0.354
	Hostility (SCL-90)	0.129	0.529
	Phobic Anxiety (SCL-90)	0.287	0.155
	Paranoid Ideation (SCL-90)	− 0.056	0.785
	Psychoticism (SCL-90)	0.006	0.979
	SCL-90 total score	0.095	0.644
	Need for Approval (ASQ)	0.279	0.095
	Difficulty in Identifying Feelings (TAS-20)	0.252	0.139
	Difficulty in describing feelings (TAS-20)	0.495	**0.002**
	Alexithimia (total score) (TAS-20)	0.341	0.042
Y-BOCS severity	Harm Avoidance (TCI)	0.094	0.580
	Self-Directedness (TCI)	− 0.143	0.399
	Drive to Thinness (EDI-2)	0.428	**0.007**
	Body Dissatisfaction (EDI-2)	0.312	0.057
	Inadequacy (EDI-2)	0.021	0.899
	Interpersonal Distrust (EDI-2)	− 0.025	0.883
	Interoceptive Awareness (EDI-2)	0.045	0.787
	Maturity Fears (EDI-2)	− 0.167	0.315
	Asceticism (EDI-2)	0.394	0.014
	Impulsivity (EDI-2)	0.115	0.491
	Social Insecurity (EDI-2)	0.220	0.184
	Somatization (SCL-90)	0.393	0.052
	Obsessive-Compulsivity (SCL-90)	0.282	0.163
	Interpersonal Oversensibility (SCL-90)	0.372	0.061
	Depression (SCL-90)	0.571	**0.002**
	Anxiety (SCL-90)	0.506	**0.008**
	Hostility (SCL-90)	0.260	0.200
	Phobic Anxiety (SCL-90)	0.377	0.057
	Paranoid Ideation (SCL-90)	0.136	0.508
	Psycoticism (SCL-90)	0.309	0.125
	SCL-90 total score	0.448	0.022
	Need for Approval (ASQ)	0.543	**0.001**
	Difficulty in Identifying Feelings (TAS-20)	0.160	0.353
	Difficulty in describing feelings (TAS-20)	0.170	0.323
	Alexithymia (total score) (TAS-20)	0.124	0.473

Accepted significance *p < *0.01

Accepted significance p < .001 is evidenced in bold characters which indicate the variables which are included in the next analysis

The Y-BOCS severity index positively correlated with drive to thinness (*p* < 0.007), depression (*p* < 0.002), anxiety (*p* < 0.008), and need for approval (*p* < 0.001).

## Discussion

In our sample, 19% of participants with AN had clinical OCD, which is consistent with the literature on the estimate of OCD prevalence in AN [49–51]. Participants with AN with a clinical OCD displayed greater symptoms severity at Y-BOCS administration. The finding of a 34% rate of participants with AN carrying severe OCD symptoms is higher with respect to the prevalence of OCD [52, 53]. This datum suggests a possible an underestimation of OCD in the AN population using clinical assessment. This may be because OCD is masked by the OC symptoms attributed to the eating symptoms. The mean total Y-BOCS in our participants with AN is very close to the average value documented by literature [23, 54, 55], and the levels of severity are not significantly different among therapeutic subgroups: this suggests a good consistence of the sample with respect to the literature.

Participants with AN displayed personality traits and eating psychopathology characteristics already documented in the literature [23, 32, 40, 41]. High values ​​of HA and low SD have been related to trait anxiety, depressive symptoms and OCD [32, 56]. Nevertheless, as it concerns the research questions, a personality link between AN and OCD symptoms is not confirmed by the present results. In fact, HA and SD scores did not correlate with Y-BOCS scores. The extensive correlation pattern of the phobic, anxiety, and depression symptoms with Y-BOCS scores was expected given that these symptoms represent a frequent comorbid psychopathology of AN [23, 24] and because of the affinity of the OCD with the anxiety spectrum [22]. It is noteworthy that obsessions and phobic anxiety were correlated. This may suggest that obsessions in women with AN may be the result of the massive expression of a coping mechanism (phobic avoidance) which underlies both phobic anxiety and food avoidance, an essential component of the pathogenesis of AN [57].

Instead, the correlation if OC symptoms of participants with AN with eating psychopathology was rather weak. Drive to thinness and asceticism are the only eating psychopathology features related to OC symptoms. AN women with OCD show a great desire to be thin, which is reached due to their high tendency to self-sacrifice which is due to asceticism [59]. Moreover both these traits and OC symptoms can be related to a common mechanism of “shifting” from inner problems to external ones [58, 60].

The hypothesis that impaired attachment and alexithymic traits could be related to OC symptoms was supported by the data. The need for approval displays the more significant and extensive correlation with the Y-BOCS dimensions. The need for approval represents the need to feel appreciated and recognized to feel confident, and it is an indicator of an insecure anxious attachment [61]. Insecure-anxious attachment is predictive of higher levels of obsessive-compulsiveness in OCD [62–64]. Moreover, an insecure attachment corresponds to greater levels of anxiety and greater vigilance on intrusive thoughts [65, 66]. Some authors suggested that insecure attachment may play a role in reinforcing distorted cognitions typical of OCD acting as a mediator between these and obsessive symptoms [63, 67]. Literature suggests that in AN women the need for approval is related to many eating psychopathology dimensions, including drive to thinness and perfectionism, to general psychopathology, and to greater obsessive-compulsiveness in particular [68]. The high need for approval appears to be the major statistical predictor of body dissatisfaction regardless of personality traits [69]. Moreover the greater need for approval, along with the “core” personality traits of AN, has been suggested as the main characteristic which differentiates AN-affected from and non-AN affected siblings living in the same family [68].

The higher levels of alexithymia in participants with AN are consistent with the literature data [70, 71]. Indeed, the present study evidences a strong relation of the difficulty in describing feelings with total OC symptoms, compulsions in particular. Even if some reports indicate that alexithymia is not always present in AN, research suggests a relevant role of this trait in predicting worse psychopathological functioning and treatment difficulties [72]. Hilde Bruch (1973) emphasizes the difficulty in subjects affected with AN in distinguishing between bodily sensations, such as hunger and satiety, and emotional tensions, as a consequence of an inadequate process of individuation-separation from the parental figures [73].

The contribution of alexithymia to the OC symptom expression can thus be linked to the evidence of an insecure attachment expressed in our participants with AN. The capacity for mentalization (i.e. the ability to interpret human behavior in terms of mental states) in today’s attachment theory derives from an appropriate maternal communication and from a mother who is sensitive with respect to the child’s experience [74, 75]. An insecure attachment would take the form of a deficit in the concept of self, which has been proposed to be the psychopathological core of eating disorders [38, 76, 77]. The self is understood as an integrated structure, which organizes and coordinates various functions (emotional, cognitive, social, motor and vegetative sensory) in relation to stimuli coming from within and from the environment [78]. People with AN would use their body image as a “proxy” of their own self since they lack of an integrated sense of self [58, 79]. A devaluation of the self as a result of the internalization of "bad" Internal Operating Models would, therefore, correspond to a dissatisfaction with one's own body [58]. An insecure attachment has been observed to be associated with a negative view of oneself, an excessive need for approval, and fear of abandonment [80].

### Strengths and limitations

Participants with AN are characterized by high levels of OC symptoms and these are consistently related with their eating and general psychopathology. Nevertheless, the roots of OC symptoms in participants with AN reveal unexpected relations. The main jointure between the AN and the OCD symptomatology does not lie neither in the common personality core nor in the eating psychopathology. The phobic-anxious features are relevant to the obsessions, but not to the compulsions. Unexpectedly, the need for approval coupled with alexithymia is suggestive of greater relevance of attachment processes for OC symptoms in AN.

Obsessive–compulsive personality, anxiety traits, and OCD are often present in childhood prior to the onset of AN and display a strong familial association [81]. AN and OCD share the strongest polygenic risk correlations from Genome Wide Association Studies (GWAS) [82]. The current findings suggest that also insecure-anxious attachment may be considered a common factor implicated in the development of both the AN [68, 69, 83, 84] and the OCD [72, 85]. OCD and AN could be alternative ways to compensate for anxious traits and insecure attachment. Further research may explore if the genetic roots of AN and DOC may represent a liability for insecure attachment itself. The regularity of compulsive rituals in OCD, as well as the severe “ascetic” control of bodily impulses in AN, could represent a maladaptive attempt to manage the fear of abandonment and the unpredictability of the relationship with the other [77, 86, 87].

Given the worse prognosis associated with the coexistence of both AN and OCD, early recognition of OC symptoms may lead to more effective treatment and better prognosis. A better understanding of the psychopathological characteristic underlying the OC symptoms may better address drug and psychotherapeutic treatments.

The AN needs a comprehensive approach (e.g. CBT-enhanced) which directly approaches relational and attachment problems [88]. It is possible that paying more attention to attachment may be useful for OCD subjects. Anyway it seems to be strategic to approach attachment troubles in those subjects who present with a comorbidity between these disorders [89]. Approaching the attachment problems by building an empathic, authoritative, stable, and caring therapeutic relationship may represent a necessary strategy to overcome the resistance to changes with both AN and OCD patients [90].

The cross-sectional nature of the study does not allow the temporal relationship between insecure-anxious attachment, obsessive–compulsive symptoms and anorexia to be explored. To confirm the association observed between the need for approval and the presence of obsessive–compulsive symptoms in the AN, a larger and more homogeneous sample of participants with AN would be preferable, and DSM-5 criteria should be applied. The group of eight women with AN and OCD in comorbidity is numerically too low to draw general conclusions about the AN-OCD population. The control group was composed of university students, and this could represent a recruitment bias. The Y-BOCS test was not administered to a control group of OCD subjects. A study of attachment dynamics which directly compares with the same assessment tools participants with AN with and without OCD, could provide further insight into the relationship between the attachment features and the psychopathological manifestations.

## What is already known on this subject?

The link between AN and OC symptoms is well-documented. AN often displays eating symptoms with OC features, and there is a frequent comorbidity between the disorders. Both disorders have been related to anxiety symptoms, but both display clear differences with respect to anxiety disorders. Finally, AN and OCD share personality traits which may represent a mediator between the two symptom clusters.

## What this study adds?

The OC symptoms in AN are underestimated if a specific assessment is not carried out. The OC symptoms of participants with AN are not strictly related to the personality traits that AN shares with OCD. The OC symptoms are independently related to attachment features, in particular the need for approval, and to phobic anxiety. The new interpretative paradigm from this finding supports a greater role of attachment abnormalities in fostering OC symptoms.

## Data Availability

Custom code.

## Declarations

### Data availability statement

The data that support the findings of this study are available from the corresponding author upon documented request.
